# Case report presenting the diagnostic challenges in a patient with recurrent acquired angioedema, antiphospholipid antibodies and undetectable C2 levels

**DOI:** 10.1186/s13223-018-0246-9

**Published:** 2018-06-04

**Authors:** Arturo J. Bonnin, Charles DeBrosse, Terri Moncrief, G. Wendell Richmond

**Affiliations:** 1 Department of Internal Medicine, Wright State University Boonshoft School of Medicine, Allergy and Asthma Centre of Dayton, 8039 Washington Village Drive, Suite #100, Centerville, Dayton, OH 45458 USA; 2 Allergy and Asthma Centre of Dayton, Dayton, OH USA; 3 Western Springs Asthma and Allergy, Western Spring, IL USA

**Keywords:** Acquired angioedema, Antiphospholipid antibody syndrome, C1 inhibitor deficiency, C2 deficiency, Lymphoma, Rituximab, Heterozygous C4 deficiency

## Abstract

**Background:**

Angioedema secondary to acquired C1 inhibitor deficiency (AAE) is a rare disease. It usually is associated with lymphoproliferative disorders. We present a case of AAE in a patient with antiphospholipid syndrome (APS), a non-Hodgkin lymphoproliferative disorder (NHL) with undetectable levels of C2, C4, and an undetectable CH50. The co-existence of AAE, APS, and NHL, with an undetectable C2 level, to the best of our knowledge, has never before reported together in the same patient.

**Case presentation:**

A patient with a recent history of thrombosis presented with recurrent episodes of angioedema. The workup revealed undetectable levels of C2, C4 and undetectable CH50. Quantitative levels of C1 inhibitor and C1q were low. C1 inhibitor function was less than 40%. Anti-cardiolipin antibodies were found. The patient was initially treated on demand with intravenous plasma-derived human C1-INH concentrates, (Cinryze^®^ Shire). Later the patient received prophylactic therapy with danazol. She was diagnosed with lymphoma 3 years after her first episode of angioedema. Single agent therapy with rituximab was not only effective in treating her lymphoma but also preventing further episodes of angioedema. Anti-cardiolipin antibody titers also declined. Additionally, marked early primary pathway complement component abnormalities and CH50 also corrected, although incomplete normalization of C4 proved to be due to a heterozygous C4 deficiency.

**Conclusion:**

This case shows the unique association of AAE, APS and NHL in a patient with undetectable levels of early complement components. Additionally, this case also shows for the first time the effectiveness of rituximab therapy in all three disease states while co-existing simultaneously in the same patient.

## Background

Angioedema secondary to acquired C1 inhibitor deficiency (AAE) presents with recurrent episodes of skin and mucosal swelling clinically indistinguishable from the hereditary form. Clinically the two are differentiated by the later onset of symptoms in AAE and the lack of family history of angioedema [1]. The prevalence of AAE has been estimated to be around 1: 600,000 [2]. The association of AAE with APS has been reported rarely [3–6], though its association preceding or following MGUS (monoclonal gammopathy of undetermined significance) and lymphomas is a common one [7]. Low serum C2 and C4 levels have been frequently found in AAE since the disease was first reported in the literature [8]. A low CH50 may be detected in any disease that involves activation of the classical complement pathway, but an undetectable CH50 suggests that a complement component is markedly depleted, functionally deficient or absent [9].

We report a case of AAE in a patient with APS with undetectable levels of complement C2, C4, and an undetectable CH50 who initially was found in a 24-h urine collection for immunofixation to have a free monoclonal kappa light chain. She was first diagnosed with monoclonal gammopathy of undetermined significance (MGUS), and eventually with NHL 3 years after her first episode of angioedema. We present in this report the serial evolution of those complement parameters with danazol and rituximab therapies and afterwards when she was in clinical remission. To our knowledge our patient is the first case reported to demonstrate the combination of these three diseases, with absence of not only C4, but also C2, coexisting in the same patient and all three conditions to be responsive to rituximab therapy.

## Case presentation

A 64 y/o Caucasian female developed pain in her left leg in December 2008. A venous ultrasound identified a thrombus. Her use of supplemental estrogen was felt to be a contributing factor. She was placed initially on enoxaparin followed by warfarin for 6 months. Thereafter she received aspirin 81 mg daily.

In January 2009, she developed mild facial and lip swelling initially attributed to facial trauma from a cat scratch. She went to the emergency department (ED) where she was treated with antihistamines and steroids and released. However, later that night she experienced oropharyngeal swelling and she returned to the emergency department. She was admitted to the intensive care unit for observation and treatment with antihistamines and systemic steroids. Evaluation by otorhinolaryngology during her admission revealed oropharyngeal edema, however, the remainder of her evaluation was unremarkable. She was discharged home 3 days later.

During the admission, an undetectable C4 level was identified. She was then referred to allergy/immunology for evaluation of angioedema and an undetectable C4 level. The family history for angioedema was negative. A review of her medication list to identify medications classically associated with an increased risk of angioedema revealed that she was taking aspirin 81 mg once daily but had not been on an angiotensin converting enzyme inhibitor. She was no longer on estrogen therapy. She had no history of diseases associated with C4 deficiency including Systemic Lupus Erythematous (SLE) and macular degeneration.

Further laboratory workup after the undetectable C4 level had been confirmed, revealed low quantitative levels of C1 inhibitor and C1q. C1 inhibitor function was less than 40%. The clinical and laboratory data suggested a diagnosis of AAE. Further complement studies revealed an undetectable level of C2 (Table 1). A C1q binding assay was positive although a C3d binding immune complex assay was negative. An initial C1q level was below the level of detection but a hemolytic C1q functional assay was normal, suggesting C1q consumption. C2 level and function were low, C3 level and function were normal, and C4 level was undetectable and function was zero (Table 2). Free and bound IgG anti-C1 esterase inhibitor measured by enzyme-linked immunosorbent assay (ELISA) was negative. An assay for IgG anti-C1q antibody was negative. Assays for IgA or IgM anti-C1 inhibitor or anti-C1q antibodies were not available (Table 3). An ANA, anti-SSA/SSB and dsDNA antibodies were negative. IgG, IgM and IgA anti-cardiolipin antibodies were positive. The titer for IgM anti-phosphatidyl serine antibody was 163.1 (normal < 22 MPS), and an IgA anti-beta 2 glycoprotein antibody level was 103.8 (normal < 20 AU). No lupus anticoagulant was detected. APS was diagnosed on March 2009.

**Table 1:**
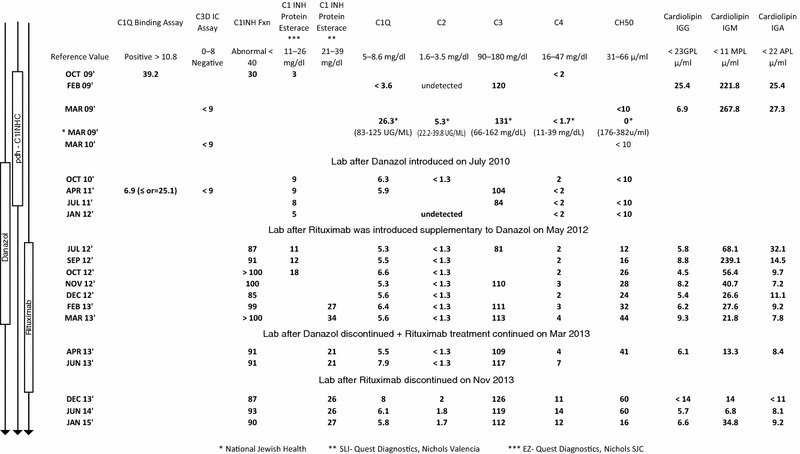
Baseline biological parameters of the patient and its changes with treatment

**Table 2:** Complement Function and Level of the Patient on March 2009

	Complement function	Reference range (units/mL)	Complement level	Reference range
C1Q	2949	2515–9414	26.3	83–125 UG/mL
C2	104	15,354–46,242	5.3	22.2–39.8 UG/mL
C3	16,725	11,249–42,887	131	66–162 mg/dL
C4	0	400,000–43,000,000	< 1.7	11–39 mg/dL
CH50	0	176–382		

Labs performed at National Jewish Health, Denver, Colorado

**Table 3:** Complement and C1 EST IHN autoantibody profile

Antibodies	Result	Reference range
C1Q autoantibody	3	[< 4.2]% of STD
C1 EST IHN—autoantibody free IgG	25.4	[0.89–39.1]% of STD
C1 EST IHN—autoantibody bound IgG	3	[0.3–46.3]% of STD

Labs performed at National Jewish Health, Denver, Colorado

Serum protein electrophoresis (SPE) with immunofixation was negative. Urine electrophoresis with immunofixation was positive for free monoclonal kappa light chains. Chest, abdominal and pelvic CT scans did not show adenopathy or organomegaly. She was referred to hematology/oncology. A bone marrow biopsy was normal on pathological analysis and flow cytometry. A positron emission tomography (PET) scan was negative. All these findings led to a diagnosis of MGUS.

**Fig. 1:**
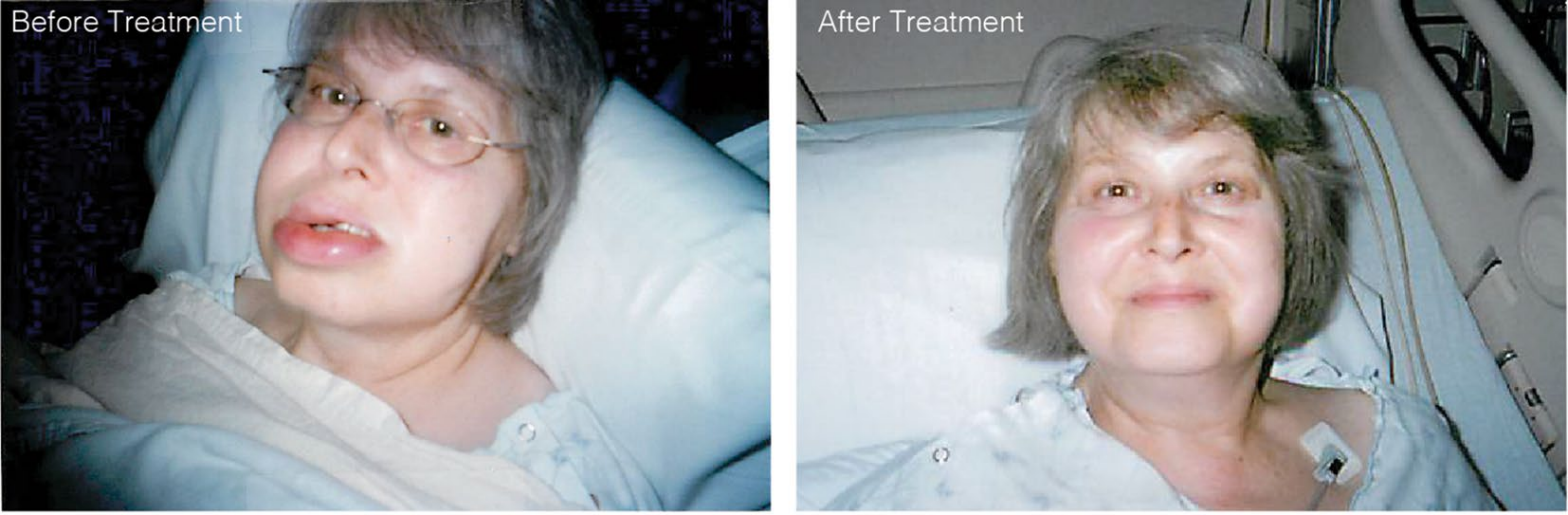
Swelling of patient before and after 24 h of treatment

The patient had five more episodes of facial and oropharyngeal edema through July 2011, each requiring hospital admission. Acute treatment with 1000 units purified pdf-C1INH (Cinryze, Shire) was effective at curtailing episodes although on two occasions, a second dose was needed (Fig. 1). Patient weighted around 81 kg during the times she received purified pdf-C1INH. After the fifth hospitalization the potential risks and benefits of prophylactic treatment were discussed with the patient. Because of her APS and recent history of DVT, it was mutually decided to initiate danazol therapy. Doses of 100 mg twice daily prevented further episodes. The dose was then decreased to the lowest effective dose of 100 mg in the morning and 50 mg at bedtime. On demand icatibant was prescribed in August 2011 when it received FDA approval but was not been administered due to success of other therapeutic interventions. Danazol was continued until March 2013.

In January 2012, she began to experience night sweats and fatigue. Her physical examination revealed the presence of splenomegaly. Her CBC revealed anemia, leukopenia, and thrombocytopenia. Her LDH and beta-2-microglobulin were elevated. Serum protein electrophoresis revealed an elevation of alpha 2 globulin fraction, with an M- band present in the gamma region. Immunofixation revealed a monoclonal IgM-Kappa gammopathy. A bone marrow biopsy was performed for the second time. It showed infiltration by monoclonal lymphocytes. Immunoperoxidase stains identified CD5—CD10+ CD20+ and BCL6+ cells, consistent with follicular lymphoma. A PET scan was done and revealed strong spleen uptake. The patient was diagnosed with low-grade B cell non-Hodgkin’s lymphoma, stage IV-B in April 2012. Treatment options were discussed with the patient. It was elected to treat her with a single agent, rituximab. Treatment was initiated in May 2012 with four doses of rituximab at 375 mg/m^2^, as initial induction therapy. By the end of the induction phase she was not experiencing night sweats or fatigue and her anemia, leukopenia and thrombocytopenia had resolved. A post treatment PET scan was negative. The patient declined another bone marrow biopsy. Following successful induction therapy, a maintenance schedule with rituximab at 375 mg/m^2^ was given.

Immunologic re-evaluation after initiation of rituximab therapy, found that her anti-cardiolipin antibodies levels decreased. The C4 quantitation remained below normal levels, although the C1 inhibitor functional assay normalized. Because of persistently decreased C4 levels, C4-genetic analysis was done. The C4 protein concentration by radio immunodiffusion assay was 13.4 mg/dL. Immunofixation of EDTA plasma detected C4A3 and C4B1 protein allotypes. Quantitative real-time polymerase reactions for complement C4 gene copy numbers performed in three independent experiments revealed: Total C4 = 3, C4A = 1, C4B = 2. These results are compatible with heterozygous C4A deficiency, suggesting a genetic component for the low levels of C4 observed in this patient. A follow-up interview with the patient in July 2016 found that she remained lymphoma free and without recurrence of angioedema or any thrombotic events.

## Discussion and conclusions

AAE association with lymphoproliferative disorders or autoantibodies is well established [10, 11]. AAE is uncommon. However, recently series have reported the presentation, associations and responses to treatment in large cohorts of patients with AAE [2, 7, 12]. This patient, in addition to having AAE, met the Sapporo Criteria for the diagnosis of Antiphospholipid Syndrome [13]. APS has rarely been reported to be associated with acquired C1 inhibitor deficiency [3–6].

The presentation of angioedema at 64 years of age in the presence of a low C4, suggested she had AAE. The detection of a quantitatively decreased C1 inhibitor in combination with decreased C1 inhibitor function was diagnostic of a C1 inhibitor deficiency. A combination of low C4 and low C1 inhibitor function has 98% specificity for C1 inhibitor deficiency and a 96% negative predictive value [14]. With the discovery that the patient had a normal SPE and a normal serum immunofixation, we entertained the possibility that the pathogenesis of her angioedema was due to an autoimmune process like her APS. The undetectable levels of C2, C4 and an undetectable CH50 suggested either a primary C2 or C4 deficiency, or less likely, a secondary deficiency due to autoantibody production and marked complement consumption [9]. Additional data revealing a low C1q level and positive C1q binding activity suggested an acquired C1 inhibitor deficiency due to anti-C1 inhibitor autoantibodies. Using an IgG- specific detection assay, anti-C1 inhibitor was not detected. The failure to detect an IgG anti-C1 inhibitor is not surprising since 30% of patients with acquired C1 inhibitor deficiency have undetectable anti-C1 inhibitor antibodies [15]. A limitation of our case presentation is that we were not able to check for IgA or IgM anti-C1 inhibitor antibodies, which can be present in AAE [7].

In an ongoing effort to evaluate for a lymphoproliferative process, a 24-h urine collection for immunoelectrophoresis and immunofixation was done. It was positive for a free kappa light chain. Further workup for a lymphoproliferative disorder was negative. Interestingly, serum immunofixation was repeated 3 years later and was found to be positive for a monoclonal IgM kappa component, suggesting that the acquired C1 inhibitor deficiency in this patient was related to a lymphoproliferative process and its M component. Previous studies have shown that the M component may have anti-C1-INH autoantibody neutralizing activity [15].

Her NHL immunophenotype was consistent with follicular lymphoma. The most common lymphoma associated with cases of AAE, is splenic marginal zone lymphoma [7, 12]. However, the most frequent type of lymphomas associated with C1 inhibitor deficiency, symptomatic or not, in a case series of patients with lymphoma was diffuse large B cell lymphoma [16].

Once the diagnosis of AAE was established, human pd-C1INHc (Cinryze-Shire) was made available for immediate administration upon this patient’s presentation in the ED. Although a dose of 1000 units was generally effective at stopping the progression of her angioedema, a second dose with 1000 units was administered within 24 h on her fourth and sixth admissions due to the persistence of lower lip swelling. Due to the recurrent nature of her angioedema episodes, we elected to try danazol. Tranexamic acid is considered to be more effective than danazol [2, 7]. Despite studies showing patients receiving danazol for > 2 years had increased coagulation [17], the known increased risk of thrombosis in patients receiving antifibrinolytics [2], influenced the decision to start the danazol trial. Danazol has been used extensively in both hereditary angioedema and AAE [18, 19] due to its ability to efficiently increase C1 inhibitor production. In our patient, danazol was well tolerated and it did prevent further episodes of AAE as long as the total dose was not lower than 150 mg/day. In addition to observe clinical benefits, the complement profile did change, similar to previous reports [19]. Later rituximab, a B cell depleting chimeric anti-CD20 monoclonal antibody [20], was used as monotherapy to treat her NHL. Although initially used in the treatment of lymphomas, it has also been used in a multitude of autoimmune diseases. Its use in APS has seldom been reported [21]. Furthermore, its administration has been reported only in individual cases of AAE patients [7, 22–25]. Its efficacy on the three coexisting conditions of our patient probably depends in that all her diseases were consequence of a monoclonal IgM kappa producing B cell lymphoproliferation.

A quantitatively undetectable C2 and essentially absent C2 function posed an interesting interpretative problem. While it is known that C2 levels are decreased in patients with C1 inhibitor deficiency [26], the finding of an undetectable C2 is not. This data strongly suggested a potential C2 deficiency, although she did not have history of recurrent pyogenic infections, SLE, or atherosclerosis commonly associated with homozygous C2 deficiency [27]. Normalization of C2 levels after treatment with rituximab ruled out a C2 deficiency and suggested a consumptive process for its markedly low level. Interestingly her C4, also undetectable initially, never normalized, suggesting a heterozygous C4 deficiency. This was confirmed by C4 genotyping studies showing that the patient had a single C4A gene and two C4B genes. Complement deficiencies have been associated with SLE and other autoimmune disorders, but are rarely reported to be associated to APS [28].

In conclusion, this patient presented with a complicated clinical course culminating in the detection of a monoclonal IgM producing lymphoproliferative disease. The pathological B cell proliferation likely was ultimately responsible for her C1 INH deficiency and APS. The complement abnormalities detected are likely multifactorial. Studies of patients with antiphospholipid antibodies have shown that these patients uniformly have significantly elevated complement activation products [29]. This, in combination with complement activation due to the intrinsic complement binding capacity of the monoclonal IgM protein or its potential role as an autoantibody, as evidenced by the acquired C1 inhibitor deficiency, lead to the uncontrolled, marked consumption of early complement components. The heterozygous C4 deficiency resulted in the expected decrease in C4 level that increased but never normalized after rituximab therapy. Prior to therapy, baseline, ‘normally’ decreased C4 levels were rapidly consumed by a monoclonal IgM kappa-associated lymphoproliferative disease causing C1 inhibitor depletion and ongoing complement consumption resulting in undetectable levels.

This case is unique for multiple reasons. To the best of our knowledge this is the first reported case of an undetectable C2 in a patient presenting with AAE. Also, this is the first report of AAE, APS and NHL co-existing in the same patient. Furthermore, simultaneous serological improvement induced by rituximab monotherapy in all three diseases has not being previously reported. Clinically her AAE and NHL improved with administration of rituximab, but it is not clear if there was clinical benefit in the patient’s APS from rituximab as previously reported [21]. She did not show any evidence of thrombotic recurrence from 2009 to 2012 in spite of no anticoagulant therapy. Interestingly, there was clinical improvement in the frequency of angioedema episodes with the administration of an attenuated androgen, danazol. This improvement was associated with a partial correction of some of the complement abnormalities, most notably an increase in C1 INH level, although never attaining a ‘normal’ level. Near complete resolution of serological and clinical findings required the correction of the monoclonal proliferative process by rituximab. This lymphoproliferative process, either alone, or concomitantly with anti-cardiolipin antibodies, drove marked complement consumption resulting in the markedly abnormal complement findings described herein.

## Abbreviations

AAE: angioedema secondary to acquired C1 inhibitor deficiency
APS: antiphospholipid syndrome
ED: emergency department
SLE: systemic lupus erythematous
PET: positron emission tomography
MGUS: monoclonal gammopathy of undetermined significance
SPE: serum protein electrophoresis
pdh-C1INH: plasma-derived human C1-INH concentrates
NHL: non-Hodgkin lymphoproliferative disorder
